# Gene Expression Profiling and Secretome Analysis Differentiate Adult-Derived Human Liver Stem/Progenitor Cells and Human Hepatic Stellate Cells

**DOI:** 10.1371/journal.pone.0086137

**Published:** 2014-01-21

**Authors:** Silvia Berardis, Catherine Lombard, Jonathan Evraerts, Adil El Taghdouini, Valérie Rosseels, Pau Sancho-Bru, Juan Jose Lozano, Leo van Grunsven, Etienne Sokal, Mustapha Najimi

**Affiliations:** 1 Université Catholique de Louvain, Institut de Recherche Expérimentale et Clinique (IREC), Laboratory of Pediatric Hepatology and Cell Therapy, Brussels, Belgium; 2 Department of Cell Biology, Liver Cell Biology Lab, Vrije Universiteit Brussel, Brussels, Belgium; 3 Liver Unit, Hospital Clínic, Centro de Investigación Biomédica en Red de Enfermedades Hepáticas y Digestivas (CIBERehd), Institut d'Investigacions Biomèdiques August Pi i Sunyer (IDIBAPS), Barcelona, Spain; University of Hong Kong, Hong Kong

## Abstract

Adult-derived human liver stem/progenitor cells (ADHLSC) are obtained after primary culture of the liver parenchymal fraction. The cells are of fibroblastic morphology and exhibit a hepato-mesenchymal phenotype. Hepatic stellate cells (HSC) derived from the liver non-parenchymal fraction, present a comparable morphology as ADHLSC. Because both ADHLSC and HSC are described as liver stem/progenitor cells, we strived to extensively compare both cell populations at different levels and to propose tools demonstrating their singularity.

ADHLSC and HSC were isolated from the liver of four different donors, expanded in vitro and followed from passage 5 until passage 11. Cell characterization was performed using immunocytochemistry, western blotting, flow cytometry, and gene microarray analyses. The secretion profile of the cells was evaluated using Elisa and multiplex Luminex assays.

Both cell types expressed α-smooth muscle actin, vimentin, fibronectin, CD73 and CD90 in accordance with their mesenchymal origin. Microarray analysis revealed significant differences in gene expression profiles. HSC present high expression levels of neuronal markers as well as cytokeratins. Such differences were confirmed using immunocytochemistry and western blotting assays. Furthermore, both cell types displayed distinct secretion profiles as ADHLSC highly secreted cytokines of therapeutic and immuno-modulatory importance, like HGF, interferon-γ and IL-10.

Our study demonstrates that ADHLSC and HSC are distinct liver fibroblastic cell populations exhibiting significant different expression and secretion profiles.

## Introduction

The liver is composed of parenchymal and non-parenchymal cell populations. Complex and well-organized interactions between such cell types allow a perfect coordination of the liver functions for preservation of the systemic homeostasis. Indeed, the liver is concomitantly managing numerous important functions such as metabolism, protein synthesis and detoxification. Hepatocytes are the main parenchymal cell type and represent the most important functional one. Liver non-parenchymal cells include epithelial bile duct cells, non-epithelial Kupffer cells, sinusoidal endothelial cells and hepatic stellate cells (HSCs) [1].

Spindle shaped HSCs are located in the space of Disse between hepatocytes and sinusoidal endothelial cells [2]. The HSC population represents about 15% of the total number of resident cells in the normal liver. These cells have several important functions including retinyl ester storage and homeostasis, remodeling of extracellular matrix, production of growth factors and cytokines, contraction and dilatation of the sinusoidal lumen [3]. During liver injury, HSC are “activated” and evolve to myofibroblast-like cells. This activation is characterized by an increase in cell proliferation and extracellular matrix protein deposition. At the structural level, activated HSC lose their big Vitamin A-containing lipid droplets and up-regulate the expression of some cell adhesion molecules like ICAM-1, VCAM-1 and NCAM and of α-smooth muscle actin as well as the secretion of pro-inflammatory cytokines [4] [5]. In vitro, part of this activation process is mimicked by culturing the cells on plastic culture dishes [6].

Our group previously obtained stem/progenitor cells from healthy adult human liver (ADHLSC). These expandable cells present a hepato-mesenchymal phenotype and have the potential to differentiate into hepatocyte-like cells both in vitro and in vivo [7] [8] [9]. Cultured ADHLSC exhibit a striking phenotypical resemblance with culture activated HSCs. Moreover, alike ADHLSCs, quiescent HSCs have been reported to express molecular markers of stem/progenitor cells and to be involved in liver regeneration [7] [10] [11].

In the current study, we carried out an extensive comparison between HSCs and ADHLSCs in order to assess the unique identity of ADHLSCs and to identify tools that can be used to differentiate both populations. To this end, we compared these mesenchymal cells after isolation from the same liver by following their phenotype, genotype and behavior in vitro from passage 5 until passage 11.

We report several characteristics similar to both cell types but shed light on significant gene expression profile and functional differences. This study confirms the unique characteristics of ADHLSCs and demonstrates their secretion potential of cytokines that could be of therapeutic and immuno-modulatory importance.

## Materials and Methods

### ADHLSC and HSC isolation and culture

The protocol and experiments were approved by the ethical committees of the St-Luc Hospital and faculty of Medicine of Université Catholique de Louvain. An agreement from the Belgian Ministry of Health was obtained for the Hepatocytes and Hepatic Stem Cells Bank. A written and signed informed consent has been obtained for each human liver used in the current study.

Four donors were used in the current study (Table 1). ADHLSC were obtained subsequently to primary culture of the liver parenchymal fraction previously obtained after a two-step collagenase perfusion, filtration and low speed centrifugation [7]. HSCs were isolated from the corresponding non-parenchymal fraction using a Nycodenz gradient centrifugation step (Myegaard, Oslo, Norway) [12].

**Table 1:** Characteristics of the four liver donors from which HSC and ADHLSC were isolated.

Donor number	Age	Gender	Reason of death	Blood group	Ischemia time
**89**	3 days	M	Respiratory	A+	4 hours
**93**	2 years	F	Metabolic disease (liver transplanted)	O+	1 hour 43
**97**	7 months	F	Meningitis	A+	5 hours 30
**98**	7 days	M	Cardio-respiratory arrest	O−	4 hours 20

Both cell types were cultured using DMEM containing 4.5 g/L glucose (Invitrogen) supplemented with 10% Fetal Calf Serum (PAA) and 1% Penicillin/Streptomycin (Invitrogen), at 37°C in a fully humidified atmosphere (5% CO2). When reaching 80% confluence, cells were lifted with 0.05% trypsin-EDTA (Invitrogen) and replated at a density of 5000 cells/cm^2^. The viability of recovered cells was evaluated using trypan blue exclusion assay.

### Immuno-cytochemistry

Cells were fixed using paraformaldehyde 3.5%, for 15 min at room temperature. Endogenous peroxidase was eliminated using hydrogen peroxide 3.3% for 3 minutes. All steps were performed at room temperature. Cells were permeabilized using D-PBS containing 1% Triton X-100 (Sigma) for 10 minutes. Non-specific immuno-staining was prevented by 1 h incubation in D-PBS containing 1% Bovine Serum Albumin (Sigma). Thereafter, cells were incubated with primary antibody for 1 h (Table 2). After washing, cells were incubated with secondary antibody (EnVision – Dako) during 30 minutes. Detection was performed after 5 minutes incubation with liquid DAB and substrate chromogen (Dako). Counterstaining was performed using Mayer's hematoxylin for 10 minutes. Preparations were then mounted for microscopic analysis (DMIL, Leica, Belgium).

**Table 2:** Primary antibodies used for the phenotypic characterization of ADHLSC and HSC by immunocytochemistry & western blotting.

Antibody	Supplier	Reference	Concentration used
ASMA	Dako	M0851	1/100
CK-18	Dako	M7010	1/350
CK-19	Dako	M0888	1/350
Desmin	Abcam	Ab6322	1/50
Fibronectin	Abcam	Ab32419	1/100
NCAM	Abcam	Ab9018	1/100
Nestin	Abcam	Ab22035	1/1000
Vimentin	Progen	105–15	1/100

### Western Blotting

Extracted Protein concentrations were determined using the BCA protein assay kit (Thermo Scientific). Fifty micrograms of protein samples were boiled for 10 min at 100°C and prepared for loading by adding dithiotreitol and bromophenol blue. Cell lysates were separated by sodium dodecyl sulfate-polyacrylamide gel electrophoresis and electroblotted onto polyvinylidene difluoride membranes. Blots were blocked with 5% milk powder in TBS with 0,2% tween (TBS-T). Following a washing step with TBS-T, membranes were incubated overnight with following primary antibodies and dilutions: CK18 (1∶350 [DAKO]), CK19 (1∶350 [DAKO]). Antibodies were diluted in blocking buffer. After additional TBS-T washes, membranes were incubated with secondary antibody for 1 h (dilution: 1/20.000). The antigens were detected by enhanced chemiluminescence using ECL substrate.

### Gene expression profile analysis

RNA was quantified with a Nanodrop1000 (Thermo Scientific) and RNA integrity evaluated with a Bioanalyzer 2100 (Agilent Technologies). 500 pg–50 ng total RNA were amplified with an Ovation Pico SL V2 Kit (NuGENE) and labelled with an Encore Biotin Module (NuGENE) following standard procedures. HG-U219 plates were hybridized with an automated array processing GeneTitan (Affymetrix).

Affymetrix gene expression data were normalized using the robust multi-array algorithm [13] using a custom probe set definition that mapped probes to 18567 Entrez Gene Ids (HGU219_Hs_ENTREZG) [14]. Genes with a coefficient of variation lower than 0.03 were eliminated, resulting in a set of 13925 genes.

For the detection of differentially expressed genes, a linear model was fitted to the data and empirical Bayes moderated statistics were calculated using the limma package from Bioconductor [15]. Adjustment of p-values was done by the determination of false discovery rates (FDR) using the Benjamini-Hochberg procedure [15]. Genes representing a change of 1.5-fold or greater and moderated p-value <0.05 were considered as differentially expressed.

Functional analysis of gene expression data was conducted using the R/Bioconductor package GOstats and the GO database (http://www.geneontology.org). Only genes that could be associated with a unique Entrez Gene ID were used. Among those, only the genes representing a fold change of 1.5 or greater and a moderated p-value<0,05 were selected. The hypergeometric distribution was used to evaluate the probability of randomly observing the enrichment for each GO term [16]. The data are deposited at Gene Expression Omnibus under reference number GSE49995.

Gene Set Enrichment Analysis (GSEA) was employed to identify biological pathways significantly associated with the ADHLSC. In comparison to other strategies for analysis of molecular profiling data that focus on high scoring individual genes, GSEA does not employ a significance threshold and evaluates microarray data at the level of gene sets defined based on prior biological knowledge. This approach has been reported to yield robust results even when dealing with heterogeneous samples with subtle sample class differences. For the current analysis, gene sets were extracted from the full Molecular Signature Database (MSigDB v.4-0). Additionally the subset of canonical pathway is analyzed alone. Analyses were based on a Signal2noise metric and a weighted scoring scheme with 1000 permutations on gene sets. Only gene sets with more than 15 genes were included in the analysis.

### RT-qPCR

Total RNA was extracted from 1.5 million cells for each cell population using Tripure isolation reagent (Roche). cDNA was generated from 1 µg of RNA using the Thermoscript™ RT kit (Invitrogen) according to manufacturer's instructions. qPCR was carried out in duplicate using TaqMan Gene expression Master Mix (Applied Biosystems) and pre-designed TaqMan probes and primers obtained from Applied Biosystems. The amplification was performed using the StepOnePlus Real-time PCR machine. The housekeeping gene cyclophilin A (PPIA) was used as a reference gene for normalization and water was used as a negative control.

### Flow cytometry

Cells were re-suspended in D-PBS at a concentration of 2×10^5^ cells/ml. For intracellular immunostaining, cell permeabilization was performed with cytofix/cytoperm for 20 minutes at 4°C (BD Pharmingen). Cells were then washed and incubated for 30 minutes at 4°C with the antibodies (see Table 3). The corresponding control isotypes were used to evaluate the non-specific binding. After washing, cells were suspended in Stabilizing Fixative (BD Pharmingen) before reading with a CANTO II flow cytometer. The analyses were performed using the BD FACSDiva Software.

**Table 3:** Antibodies used for the phenotypic characterization of ADHLSCs and HSCs by flow cytometry.

Antibody	Fluorochrome	Corresponding isotype	Supplier	Reference	Concentration
Anti-ASMA	FITC	MsIgG2a	Abcam	Ab8211	1/10
Anti-CD29	APC	MsIgG1, k	BD	559883	1/10
Anti-CD44	FITC	MsIgG2b, k	BD	555478	1/10
Anti-CD45	PE-Cy7	MsIgG1, k	BD	557748	1/10
Anti-CD73	PE	MsIgG1, k	BD	550257	1/10
Anti-CD90	APC	MsIgG1, k	BD	559869	1/10
Anti-CD117	APC	MsIgG1, k	BD	550412	1/10
Anti-CD133/2	PE	MsIgG1	Miltenyi	130-080-901	1/5

### ELISA

When 60–70% of confluence was reached, the cells were washed and the conditioned medium was replaced by a serum-free medium. After 24 hours incubation, supernatants were collected and cells were lifted for counting and viability evaluation using trypan blue exclusion assay.

For the collagen secretion analysis, we used an Elisa kit for the procollagen type I C-Peptide (Takara Bio Inc, Japan). Hepatocyte Growth Factor (HGF) and Transforming Growth Factor beta 1 (TGFβ1) levels in the culture supernatants were assayed by using Quantikine Elisa Kits from R&D Systems. The experiments were performed according to the manufacturer's instructions. For TGFβ1 Elisa, samples were activated by acidification followed by neutralization in order to activate latent TGFβ1 to immune-reactive TGFβ1 detectable by the Quantikine TGFβ1 immunoassay. The measurement of the absorbance at 450 nm was done with a Victor X4 plate Reader (PerkinElmer). For HGF and TGFβ1 kits, a reading at 570 nm was subtracted to the 450 nm reading to correct the optical imperfections of the plates.

### Luminex analysis

We also used a 27plex kit (IL-1b, IP-10, IL-2, IL-4, IL-6, IL-7, IL-9, IL-10, IL-13, IL-15, Eotaxin, FGF, GM-CSF, INF-γ, MIP-1a, MIP-1b, RANTES, TNFα, IL-1ra, IL-5, IL-8, IL-12, IL-17, G-CSF, MCP-1, PDGF-bb and VEGF) and the Luminex technology (Bio-Plex 200, Biorad) to investigate the secretome of both liver cell types. The principle of the technique is based on color-coded beads and enables to detect up to 100 cytokines simultaneously. The primary antibody directed against the target protein is conjugated with the dyed beads. After several washes to remove unbounded proteins, a secondary biotinylated antibody is added to the reaction. Streptavidin-phycoerythrin (Streptavidin-PE) is then added to bind the biotinylated antibody. By measuring the relative fluorescence intensity, the antigen-antibody reaction can be measured. The assays were performed following the manufacturer's instructions.

Briefly, after the pre-wetting of the plate, 50 µl of the beads were added in each well and washed twice. 50 µl of the samples (serum free culture supernatants recovered after 24 hours of culture) were added to the plate. The plate was shaken during 30 seconds and then incubated for 45 minutes on a plate shaker at 120 rpm at room temperature. The plate was washed three times with the Bio-Plex wash buffer and 25 µl of the detection antibody was added in each well and incubated for 30 minutes on a plate shaker at 120 rpm. The plate was then washed three times with the Bio-Plex wash buffer and 50 µl of the Streptavidin-PE solution was added in each well. The plate was shaken during 30 seconds and incubated for 10 minutes on a plate shaker at 120 rpm. Finally, after three washes of the plate with the Bio-Plex wash buffer, the beads were resuspended with 125 µl of Bio-Plex Assay Buffer. The plate was read by the Luminex machine and the data were analyzed using Bio-Plex Manager 6.0.

### Statistics

Results, others than those of microarray studies, are expressed as mean ± standard error of the mean (SEM). Statistical differences were determined by Student's *t* test for two groups' comparison. Differences were considered significant when p values *p<0.05, **p<0.01, ***p<0.001.

## Results

### Phenotypic and genotypic characterization of ADHLSC and HSC

**Figure 1:**
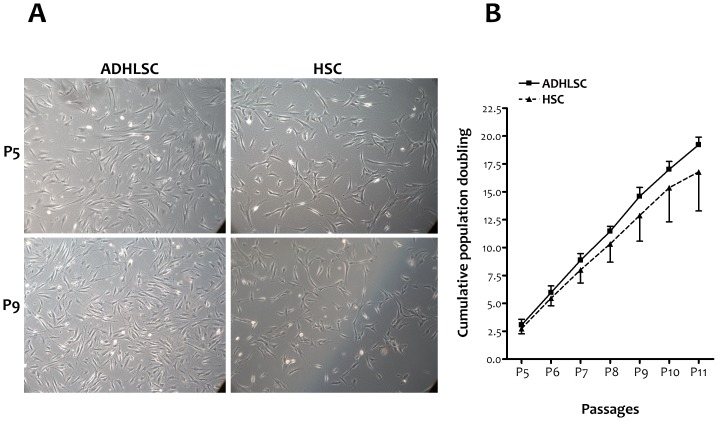
ADHLSC and HSC in culture. *A*, Fibroblastic morphology at passages 5 & 9 as shown using phase contrast microscopy, original magnification 100×. *B*, Cumulative population doubling of both cell types from passage 5 to passage 11 (n = 4).

For each liver donor, HSC and ADHLSC were cultivated under the same culture conditions and concomitantly followed. The fibroblastic morphology displayed by both cell types remained stable over the different studied passages (Figure 1A). The population cumulative doubling was similar for the two cell types (Figure 1B).

We investigated the mesenchymal phenotype of both cell types (n = 4) by exploring the expression of several specific appropriate markers using flow cytometry. Both cell types were immuno-positive for most of the membrane markers widely used to characterize mesenchymal stem cells. This was the case for CD73, CD90, CD29, and CD44 for which expression levels remained stable for the analyzed passages (Table 4). No significant difference was noticed between HSC and ADHLSC. These findings were confirmed using immunocytochemistry and RT-qPCR after analyzing the expression of other classical mesenchymal markers like vimentin, α-smooth muscle actin and fibronectin (data not shown). The mesenchymal phenotype of both cell types was also supported by the negative expression of hematopoietic markers like CD45, CD117 and CD133 as demonstrated using flow cytometry (Table 4).

**Table 4:** Phenotypic characterization of ADHLSC and HSC by flow cytometry for mesenchymal stem cells, hematopoietic cells and extracellular matrix markers.

				ADHLSC		HSC	
				P5	P11	P5	P11
**Mesenchymal stem cells markers**							
			*ASMA*	87,8±4,7%	83,8±2,9%	91,9±5,5%	87,4±12,6%
			*CD73*	88,2±5,9%	90,2±6,1%	89,9±2,5%	82,8±20,1%
			*CD90*	97,2±2,7%	92,7±7,8%	95,6±2,3%	88,7±10,4%
**Hematopoietic cells markers**							
			*CD133*	0,7±0,2%	1,3±0,3%	2,1±2,2%	2,2±2,3%
			*CD45*	1,4±0,8%	2,6±3,1%	1,7±1,0%	1,8±1,9%
			*CD117*	0±0%	0,07±0,12%	0,3±0,4%	0,03±0,06%
**Extracellular matrix markers**							
			*CD49b*	95,6±1,1%	95,5±2%	89,7±8,0%	90±9,7%
			*CD29*	86,4±10,2%	60,8±11%	77±21,5%	56,6±49,64%
			*CD44*	77,5±8,6%	79,7±9,2%	68±4,1%	75±18,9%

Data presented as mean percentage of immunopositive cells from four different donors at early (P5) and late(P11) passages. No significant difference was observed between the two cell populations.

**Figure 2:**
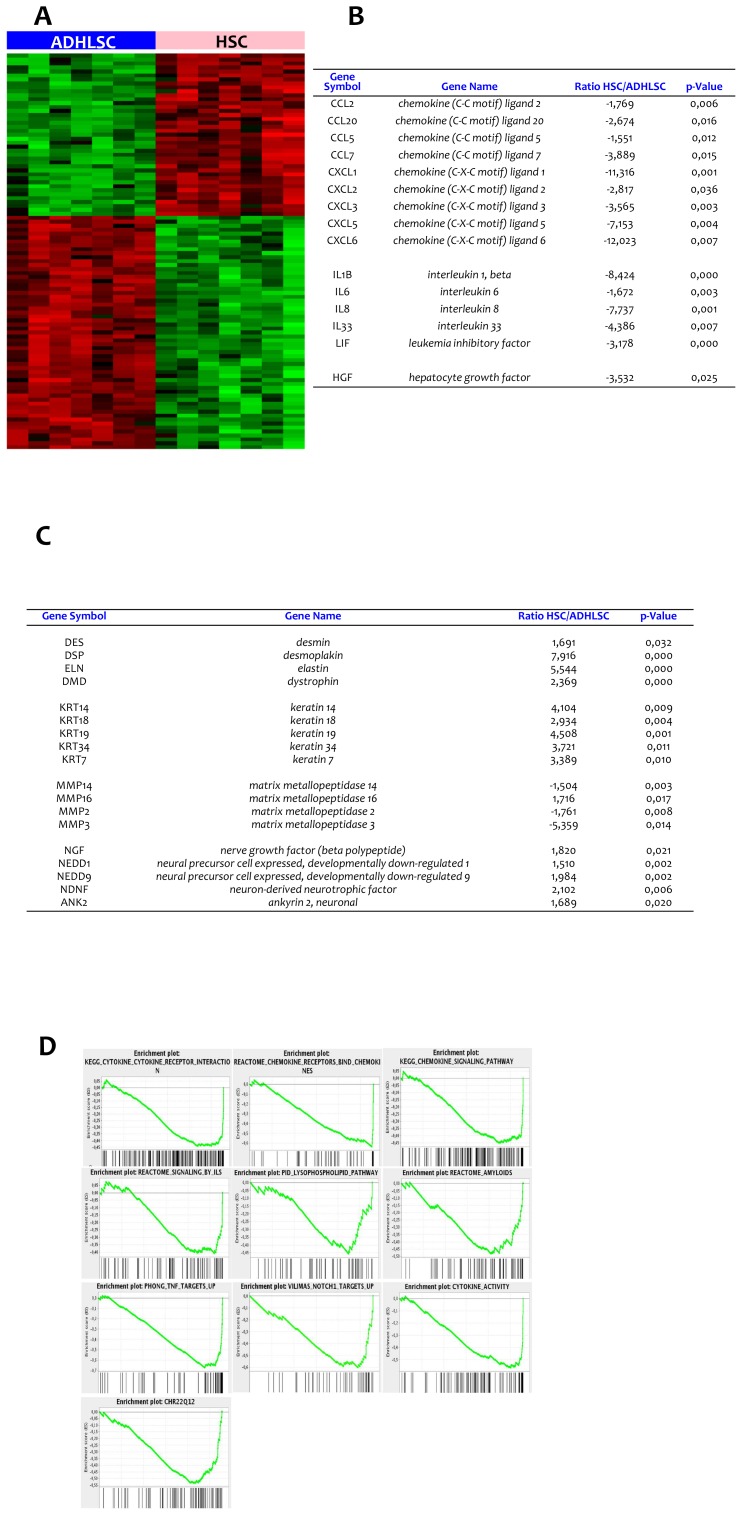
Gene expression profile analysis of ADHLSC and HSC using microarrays. *A*, Hundred top genes significantly and differentially expressed in the two cell populations (4 samples from passage 5 for all donors and 3 samples at passage 7 for 3 donors including the diseased donor). Red bars denote over-expression, black bars denote equally expression and green bars denote under-expression. B, chemokines and cytokines expression profile. C, intracytoplasmic, intermediate filaments, and neural growth factors. D, Gene Set Enrichment Analysis (GSEA). Selected statistically significant GSEA plots obtained from enriched ADHLSC. Within these plots, the green line represents the sliding enrichment score and the black bars demarcate the position of the gene set members within the ranked expression data.

This first comparative characterization only confirmed the mesenchymal phenotype of ADHLSCs and HSCs but did not allow us to discriminate both cell populations. We thereafter studied the gene expression profile of ADHLSCs and HSCs using Affymetrix HG-U219 gene chips expression. The analysis was performed on 7 different samples for each cell population (4 samples from the four donors at Passage 5+3 samples from only 3 donors at Passage 7). These experiments allowed us to point out significant signature differences (100 top genes that are significantly differentially expressed in the two cell populations) after screening of 13925 genes (Figure 2A). We noticed that liver stem/progenitor cells highly and predominantly expressed chemokine ligands such as CXCL1, CXCL3, CXCL5, CXCL6 and CXCL7 when compared to HSCs (Figure 2B). ADHLSCs also displayed increased levels of cytokines like IL-1β, IL-6, IL-8, IL-33, and LIF as well as of growth factors like HGF in comparison with HSC (Figure 2B). Regarding cytoplasmic markers, HSCs expressed significantly higher amounts of desmin (type III intermediate filament), elastin, desmoplakin (anchors intermediate filaments to desmosomal plaques) and dystrophin as compared to ADHLSC (Figure 2C).

When comparing MMP expression between the two cell types, we noticed an elevated expression of MMP2, MMP3 and MMP14 in ADHLSCs and an increased expression of MMP16 in HSCs (Figure 2C).

In order to obtain a functional annotation of our gene expression results, we used the Gene Set Enrichment Analysis (GSEA) method. We have used GSEA to identify those most relevant “canonical” pathways and Gene ontology categories enriched in ADHLSC. Results are included in Figure 2D and Table 5.

**Table 5:** Gene Set Enrichment Analysis biological: Pathways significantly associated with ADHLSC and the corresponding FDR as well as the enriched genes.

GENESET	SIZE	FDR_P VALUE	ENRICHED GENES
KEGG_CYTOKINE_CYTOKINE_RECEPTOR_INTERACTION	213	0,000	CXCL5 CXCL1 CSF3 IL1B CXCL3 CXCL6 BMP2 IL8 CCL7 HGF CSF2 IL11 CCL20 LIF CCL11 TGFB1 TNFRSF19 CXCL2 IL24 IL1A CCL5 TNFRSF21 CCL8 CLCF1 EPOR CRLF2 IL19 IL15RA CCL24 TNFRSF10A RELT CNTFR FLT3 TNFRSF12A TGFB2 IL17RA CSF1 TNFRSF1A VEGFA CXCR3 AMHR2 AMH TNFRSF10B CCL23 CCL2 TGFBR2 TNFRSF1B CCL26 IL6 CCL3 IL7R CCR9 LTB IL18R1 TNFSF15 IL17RB EPO NGFR CCL4 CD40 TNFSF4 CXCL10 PDGFB IL10RB TNFSF8 CX3CL1 ACVR1B CD27 IL2RG TNFRSF8 FLT4 IFNE TNFSF14 TNFRSF10C CCR7
REACTOME_CHEMOKINE_RECEPTORS_BIND_CHEMOKINES	46	0,000	CXCL5 CXCL1 CXCL3 CXCL6 IL8 CCL7 CCL20 CCL11 CXCL2 CCL5
KEGG_CHEMOKINE_SIGNALING_PATHWAY	135	0,002	CXCL5 CXCL1 CXCL3 CXCL6 IL8 CCL7 CCL20 GNB2 CCL11 RAC2 CXCL2 CCL5 CCL8 PRKX PIK3CD TIAM2 SHC1 CCL24 GNAI2 PIK3R2 PREX1 CXCR3 BCAR1 NFKBIA CCL23 CCL2 CCL26 GNG2 NFKBIB CCL3 CCR9 PRKACA IKBKG CSK ADRBK1 CCL4 PXN PRKCZ CXCL10 STAT2 CX3CL1
REACTOME_SIGNALING_BY_ILS	70	0,041	IL1B IRAK3 HGF CSF2 IL1A PIK3CD SOCS3 SHC1 NFKB2 IRAK2 TAB1 PIK3R2 CASP1 GAB2 PELI3 IL6 IL7R IKBKG MYD88 IRAK1 SQSTM1 PELI2 MAP2K4 NOD2 IL2RG
PID_LYSOPHOSPHOLIPID_PATHWAY	51	0,028	IL8 GNA11 TRIP6 GNAI2 MMP2 ARHGEF1 BCAR1 NFKBIA PLCG1 GNG2 IL6 HBEGF FOS GNAZ JUN ADRA1B PRKCE PXN GNA12 PLD2 ADCY5
REACTOME_AMYLOIDS	59	0,009	HIST1H3I HIST1H2BM HIST1H2BN CST3 HIST1H4K HIST4H4 HIST1H3F HIST1H2BL SNCA HIST1H3B HIST1H2BF HIST1H3A HIST3H2BB HIST1H2BH HIST1H2BG HIST2H2BE HIST1H4L HIST1H3D HIST1H2BC HIST1H4D HIST1H3E HIST1H3G HIST1H3J HIST1H2BE HIST1H2BO HIST1H2AJ HIST1H2BI HIST1H4B HIST1H4H MFGE8
VILIMAS_NOTCH1_TARGETS_UP	40	0,001	BCL2A1 ICAM1 CCL5 HEY1 JUNB EGR1 NFKB2 THY1 EGR2 RELB BIRC3 DTX1 CARD11 NFKBIA ZAP70 P2RY10
PHONG_TNF_TARGETS_UP	56	0,000	CXCL1 CXCL3 BMP2 IL8 CSF2 IL11 CCL20 LIF ICAM1 CXCL2 ETS2 PLAU IER2 LDLR TNFAIP3 IRF1 BTG1 JUNB EGR1 NFKB2 NKX3-1 ATF3 EGR2 BIRC3 DUSP1 NFKBIA TNFRSF10B REL IL6 FOS SDC4 JUN MCL1
CHR22Q12	74	0,000	TBC1D10A RAC2 APOL2 SMTN HMOX1 PES1 SUN2 MAFF SELM EMID1 PIK3IP1 TOB2 CSNK1E GATSL3 CBX6 PISD SEC14L2 MYH9 SLC16A8 GAS2L1 GALR3 PDXP APOL5 CACNA1I TOMM22 RBFOX2 PVALB ASCC2 CYTH4 MTMR3 SYN3 HORMAD2 SLC5A4 PDGFB CHEK2 OSBP2 DMC1 APOL6 KREMEN1
CYTOKINE_ACTIVITY	94	0,000	CXCL5 CXCL1 CSF3 CXCL3 CXCL6 IL8 CCL7 CSF2 CCL20 CCL11 CXCL2 CCL5 CCL8 GDF15 NAMPT IL19 TRIP6 CCL24 CDK5 TGFB2 CSF1 VEGFA MIF CCL23 CCL2

**Figure 3:**
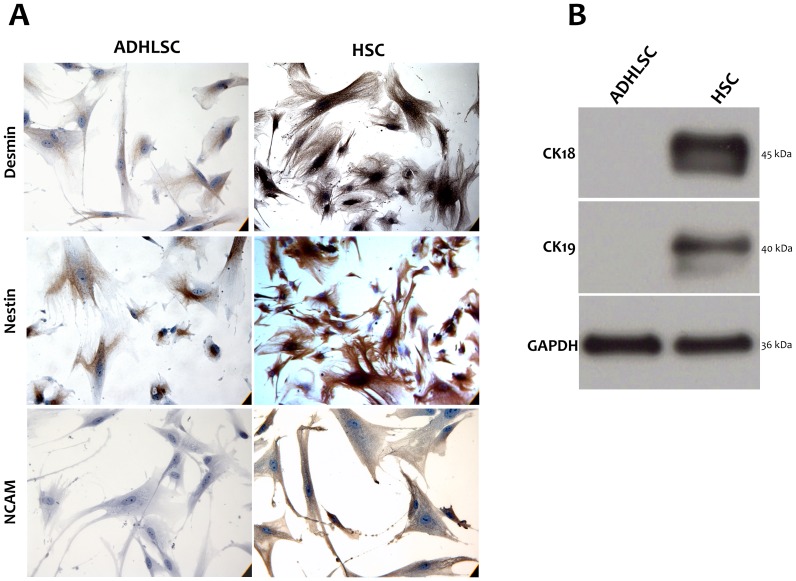
Immunodetection comparative study. A, HSCs show a positive immunostaining for neural markers like NCAM and nestin and for desmin. The expression level of nestin and desmin is higher in HSC in comparison with ADHLSC. ADHLSC do not express NCAM at the protein level. Images are representative of several fields examined from four different donors. Magnification 400×. B, Total proteins were extracted from ADHLSC and HSC and CK-18 and CK-19 immuno-detection was realized using western blotting. Data shown are representative of 4 different cell samples isolated from 4 different donors.

Interestingly, HSCs exhibited significantly higher levels of neuronal markers such as NGF, neurotrophin 3, NDNF, and NEDD 2 & 9 than ADHLSCs (Figure 2C). Such significant differences were also confirmed using immuno-cytochemistry. Indeed, we observed that NCAM is expressed in HSC but not in ADHLSC; a difference stably maintained over the passages (Figure 3A). Nestin and Desmin expression was predominant in HSCs as compared to ADHLSC (Figure 3A).

HSC but not ADHLSC expressed hepatic cytokeratins like cytokeratin 18 and cytokeratin 19 at the protein level, as demonstrated using western blotting (Figure 3B). These observations are in accordance with the microarray analyses that revealed a higher expression level of keratins 7, 14, 18, 19, & 34 by HSC when compared to ADHLSCs (Figure 2C).

All together, these findings clearly show that cultured HSCs and ADHLSCs are two different cell populations with distinct gene expression profiles.

### Functional characterization of ADHLSCs and HSCs – Cytokines and growth factors secretion

**Figure 4:**
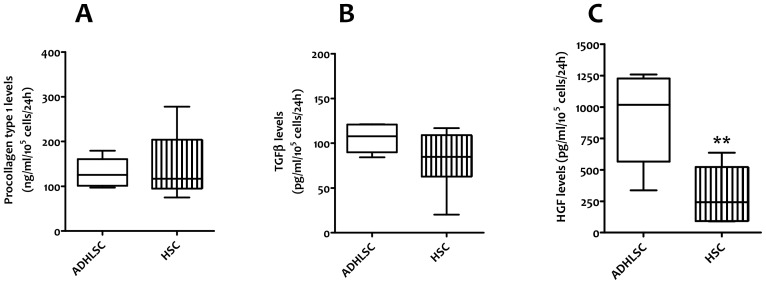
Secretome profile in the conditioned culture medium of ADHLSC and HSC. The Elisa analyses were performed on supernatants (n = 3 including the diseased donor, analyzed at passage 5 and passage 7) collected 24 hours after incubation with serum free medium. *A*, the collagen secretion was evaluated by measuring the procollagen type-I C-Peptide, precursor of the collagen type I, by Elisa. No significant difference was observed between HSC and ADHLSC. *B*, TGFβ1 secretion; ADHLSC and HSC secrete an equivalent amount of TGFβ1 in the culture supernatant. *C*, HGF secretion; we observed a significant difference in HGF secretion between HSC and ADHLSC. The liver stem/progenitor cells seem to secrete HGF about three times more than HSC.

After phenotypic and genotypic characterization, we carried out a functional analysis of both cell populations by assessing their secretome profile in the corresponding conditioned culture medium of cells originated from 3 different donors at passages 5 &7. This was performed on supernatants collected 24 hours after incubation with serum free medium. As collagen is known to be secreted by activated HSCs, we evaluated its secretion by measuring the pro-collagen type-I C-Peptide in the culture supernatants. No significant difference was observed between HSCs and ADHLSCs (Figure 4A). We thereafter looked at some markers known to play important functional roles in terms of immuno-modulation and liver regeneration. Secretion of Transforming growth factor-beta 1 (TGF-β1), one of the most powerful pro-fibrotic cytokines and involved in inflammatory and immune responses, was confirmed in our activated human hepatic stellate cell cultures. We demonstrated that ADHLSCs secrete equivalent amounts of TGFβ1 in the culture supernatant (Figure 4B).

HGF is a hepatocyte mitogen, which has several crucial physiological functions including organ protection and regeneration. Following liver injury, HGF is known to be secreted by distant organs such as spleen, lungs and kidneys as well as by sinusoidal cells such as Kupffer cells and HSCs. Moreover, HGF has anti-inflammatory properties. We observed a significant difference in HGF production between HSCs and ADHLSCs. The liver stem/progenitor cells seemed to secrete HGF about three times more than HSCs (Figure 4C).

**Figure 5:**
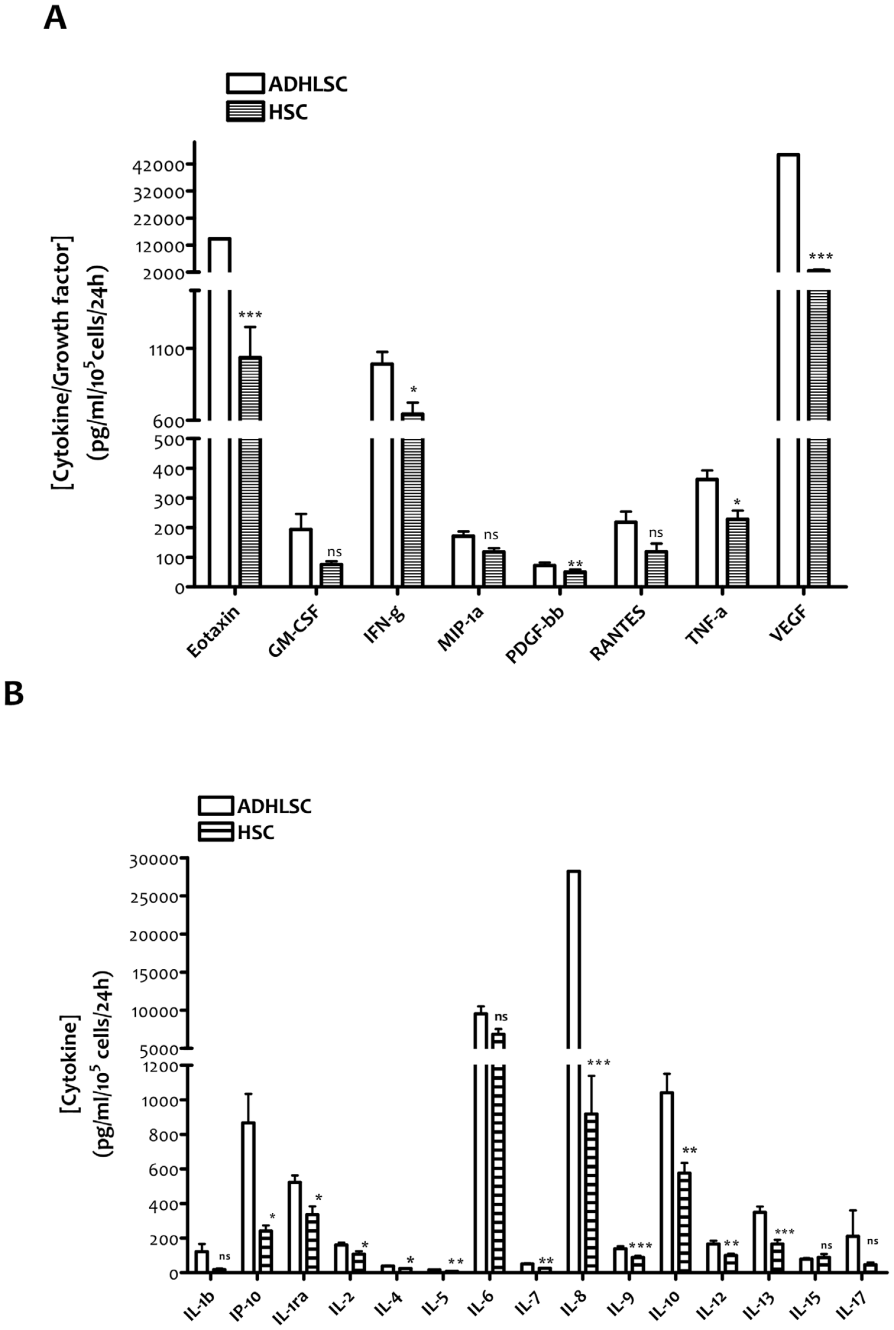
Secretome analysis of ADHLSC and HSC using a multiplex technology (Luminex). The analyses were performed on culture supernatants collected 24(n = 3, from 2 healthy and 1 diseased donors; analyzed at passage 5 and passage 7). The growth factors and cytokines concentrations were calculated for 100 000 cells. *A*, ADHLSC secrete high levels of VEGF, PDGFbb and Eotaxin as compared to HSC. *** denotes a p value <0,001; ** denotes a p value<0,01; * denotes a p value<0,05. NS: Not significant. *B*, concerning pro-inflammatory cytokines, ADHLSC highly secreted IP-10 (3.6 times), IL-5 (2 times), IL-7 (2 times), IL-8 (30 times), and IL-17 (4.5 times) as compared to HSC. With respect to anti-inflammatory cytokines, ADHLSC significantly secreted higher level of IL-13 (2.1 times) and IL-10 (1.8 times). *** denotes a p value<0,001; ** denotes a p value<0,01; * denotes a p value<0,05. NS: Not significant.

In the second part of this functional analysis, we focused on cytokines implicated in the inflammatory response. Indeed, HSC are known to secrete several pro-inflammatory cytokines while ADHLSC seem to exhibit immuno-modulatory properties. Twenty-seven cytokines were analyzed, using a multiplex technology. The cytokines were classified as growth factors, chemokines, pro-inflammatory cytokines, anti-inflammatory cytokines and those with dual roles. Among the growth factors analyzed, the main differences were noticed for VEGF and PDGFbb for which significant higher levels were detected in ADHLSCs (Figure 5A). The increase in VEGF and PDGFbb levels was respectively 17 and 1.5 times as compared to HSCs from the same donor. Regarding the chemokines analyzed, Eotaxin (CCL11) was 14 times more secreted by ADHLSC than HSC (Figure 5A). With respect to pro-inflammatory cytokines, the major differences between the two cell populations were IP-10 (3.6 times), IL-5 (2 times), IL-7 (2 times), IL-8 (30 times), and IL-17 (4.5 times), which are highly secreted by ADHLSC. To a lesser extent, the same trend was observed for IL-9 (1.6 times), IL-12 (1.7 times), interferonγ (1.5 times) and TNFα (1.6 times) (Figures 5A & 5B). Concerning the anti-inflammatory cytokines, a significant higher level of IL-13 (2.1 times) and IL-10 (1.8 times), was secreted by ADHLSCs as compared to HSCs. IL-1ra, a natural inhibitor of the pro-inflammatory effect of IL1β, and IL-4 (presenting anti- and pro-inflammatory properties) were also produced in higher concentrations by ADHLSCs, even if the statistical difference was less important (Figure 5B). IL-2 is considered to play a dual role as it has pro- and anti-inflammatory activities. The concentration of this cytokine was found to be higher in the culture supernatant of ADHLSCs (1.5 times) in comparison with the one of HSCs (Figure 5B).

These findings confirm the singularity of both cell populations and support the expression profile differences demonstrated using gene expression analyses.

## Discussion

The aim of the current study was to establish an in-depth comparison of ADHLSCs and HSCs, cells of human liver origin exhibiting similar fibroblastic morphology and both reported as stem/progenitor cells. By comparing these cell populations successfully isolated from the same donors, we i) demonstrate that ADHLSC and HSC are two distinct liver cell types displaying significant differences in gene expression profiles as well as functional features, and ii) confirm the stem/progenitor properties of ADHLSC.

Using flow cytometry and immuno-cytochemistry, we confirmed the mesenchymal phenotype of both ADHLSCs and HSCs. Both cell populations were also negative for hematopoietic and epithelial protein markers rendering the demonstration of their singularity difficult.

To assess a thorough analysis of both cell types, we used microarray analysis and screened more than 10.000 genes on the 4 different samples of ADHLSC and HSC. Top 100 differentially expressed genes were identified. The main differences between ADHLSCs and HSCs are seen at the level of the chemokines expressed. Such chemotactic molecules which regulate the infiltration of immune cells to sites of inflammatory injuries, are initially all expressed in the liver. With respect to the first family, CCL7 seems to be the highly expressed one in ADHLSC in accordance with its documented role in development as demonstrated in neuronal differentiation [17]. CCL7 was also involved in the pro-inflammatory responses that may stimulate liver regeneration at the cellular level [18].

The second CXC chemokine family displays well-documented neutrophil chemotactic, angiogenic, and mitogenic properties [19] [20]. The increased expression levels of CXCL1 and CXCL6 detected in ADHLSC are in accordance with the parallel up-regulated level of IL-8 and LIF. All these cytokines are directly involved in chemotaxis, cell movement and migration of mesenchymal stem cells [21].

HGF, a pleitropic cytokine of mesenchymal origin, participates in the regulation of proliferation, differentiation and chemotactic migration of mesenchymal stem cells [22] [23]. Its high expression level in ADHLSCs is in accordance with their stem/progenitor features as documented in other MSCs. Conversely, HGF levels are abnormally decreased in fibrosis settings which correlate with induced proliferation of activated stellate cells [24]. In the same context, HGF over-expression induces a remarkable anti-fibrotic effect [25] [26]. The anti-fibrogenic effects of HGF have been attributed to the inhibition of the TGFβ pathway, the inhibition of other fibrogenic cytokines and to the induction of matrix metalloproteinases [27] as well as up-regulation of miR-29 thereby regulating collagen expression [28]. This result may suggest potential anti-fibrotic properties of ADHLSC as demonstrated for other mesenchymal stem cells [29] [30].

In our study, we did detect protein expression of cytokeratins 18 & 19 in only HSC as demonstrated using western blotting which is in accordance with gene expression profiling data. The data are supported by the increased expression of desmoplakin, which anchors these intermediate filaments to desmosomes [31]. However, no data is currently available regarding desmoplakin expression and its role in activated HSCs.

After its interaction with dystroglycan, dystrophin forms a glycoprotein transmembrane complex required for spatial organization of laminin on the cell surface and for basement membrane assembly. Such complex is up regulated during spontaneous activation of HSC in culture [32]. This is also the case for elastin, a marker of maturity of liver fibrosis that was logically up regulated in HSC as compared to ADHLSC [33].

MMP3 (stremolysin), which degrades collagen types II, III, IV, IX, and X, proteoglycans, fibronectin, laminin and elastin, was the highly up-regulated MMP in ADHLSC compared to others MMPs like MMP2 and MMP14. In contrast, MMP3 is down-regulated in other MSCs like adipose-tissue MSC, as compared to fibroblasts [34]. HSCs are known to express both mesenchymal and neural cell lineage markers [35]. Indeed, an increasing number of neural/neuroectodermal markers have been documented in HSCs like NCAM, nestin and NGF [36] [37] [38]. In our study, we confirmed this specificity of HSCs using both microarray and immunocytochemistry. At the protein level, we demonstrated that NCAM is highly expressed in HSCs as compared to ADHLSC (although no gene expression level difference was seen between both cell types). Nestin expression presents the same profile as NCAM except that its protein expression is detectable in ADHLSC. Indeed, this intermediate filament has been described in mesenchymal stem/progenitor cells [39]. These data are in accordance with the described genetic profile of other MSCs, for which neural markers are down-regulated in comparison with fibroblasts [34]. Together, these phenotypic and genotypic characterization analyses confirmed the singularity of ADHLSCs and HSCs with respect to chemokines, intermediate filaments and neural markers expression levels.

The functional analysis of ADHLSCs and HSCs allowed us to support the hypothesis that both cell populations also behave differentially in basal conditions. Obviously, the secretion profile of each cell type is different and supported the microarray data, emphasizing that ADHLSCs secrete cytokines of therapeutic and immuno-modulatory importance.

In the liver, TGF-β is initially produced in non-parenchymal liver cells, whereas absent in fully differentiated epithelial cells [40]. TGF-β is also a potent activator of myofibroblast differentiation, ECM synthesis, migration, and oxidant production in mesenchymal cells [41]. TGF-β activates HSC, stimulates their ECM secretion and inhibits their apoptosis, which is in accordance with its pro-inflammatory effects [37]. We recently demonstrated the immuno-modulatory effects of ADHLSC and TGF-β may participate to these effects by inhibiting lymphocytes proliferation, macrophages activation and immunoglobulins secretion [42] [43].

Collagen is one of the predominant structural proteins of the liver extracellular matrix and is involved in stem cell proliferation and differentiation [44] [45] [46]. Accordingly, a comparable secretion level was measured in both analyzed cell types. We also focused on cytokines implicated in the immune response and demonstrated that ADHLSC secreted greater amounts of pro-inflammatory cytokines like IL-7, IL-8, IL-9, IL-12, interferonγ and TNFα and of some anti-inflammatory cytokines like IL-1ra, IL-4, IL-10 and IL-13. All these cytokines are secreted by adipose-derived MSC, stem cells possessing interesting immuno-modulatory properties [47], a feature recently also demonstrated in ADHLSC [42]. In addition, interferon-γ, even with pro-inflammatory properties, has been described as an inhibitor of collagen deposition and HSC activation in vitro and in vivo [48].

These secretion profile analyses also suggest that ADHLSC secreted cytokines and growth factors may be of therapeutic and immuno-modulatory importance in the context of their transplantation development.

VEGF has been shown to promote liver fibrosis by stimulating the activation, proliferation, and chemoattraction of HSCs [49] [50] [51]. In our study, we demonstrated that VEGF is highly secreted in ADHLSCs as compared to HSCs, which is in accordance with its involvement in wound healing [52] and regeneration process including for the liver [53] [54] [55]. VEGF has also been documented to be involved in supporting stem cell proliferation and survival [56].

In conclusion, our study demonstrates that ADHLSCs and HSCs represent two distinct hepatic cell populations. Even if they share numerous similar characteristics, we highlighted significant differences supporting their singularity mainly at the gene expression and secretion profile levels. This study also provides additional features supporting the potential therapeutic development of ADHLSC for liver cell-based therapy.
